# Phenotypic correlation between queen and worker brood care supports the role of maternal care in the evolution of eusociality

**DOI:** 10.1002/ece3.4475

**Published:** 2018-10-03

**Authors:** Justin T. Walsh, Lisa Signorotti, Timothy A. Linksvayer, Patrizia d'Ettorre

**Affiliations:** ^1^ Department of Biology University of Pennsylvania Philadelphia Pennsylvania; ^2^ Laboratory of Experimental and Comparative Ethology (LEEC) University of Paris 13 Sorbonne Paris Cité France

**Keywords:** brood care, caste, eusociality, heterochrony, maternal care

## Abstract

Cooperative brood care by siblings, a defining feature of eusociality, is hypothesized to be evolutionarily derived from maternal care via shifts in the timing of the expression of genes underlying maternal care. If sibling and maternal care share a genetic basis, the two behaviors are expected to be genetically and phenotypically correlated. We tested this prediction in the black garden ant *Lasius niger* by quantifying the brood retrieval rate of queens and their first and later generation worker offspring. Brood retrieval rate of queens was positively phenotypically correlated with the brood retrieval rate of first generation but not with later generation workers. The difference between first and later generation workers could be due to the stronger similarity in care behavior provided by queens and first generation workers compared to later generations. Furthermore, we found that queen retrieval rate was positively correlated with colony productivity, suggesting that natural selection is acting on maternal care. Overall, our results support the idea of a shared genetic basis between maternal and sibling care as well as queen and worker traits more generally, which has implications for the role of intercaste correlations in the evolution of queen and worker traits and eusociality.

## 1 INTRODUCTION

Sibling care, in which social insect workers forego reproduction to care for their younger siblings, is one of the defining characteristics of eusociality (Wilson, 1971). Ultimately, the evolution of sibling care can be explained by kin or colony‐level selection (Boomsma, 2007; Hamilton, 1964; Lehmann & Keller, 2006; Wade, 1985; Wilson & Holldobler, 2005). However, the proximate mechanisms underlying sibling care are less understood. A series of hypotheses suggest that sibling care is evolutionarily derived from maternal care, so that maternal and sibling care are expected to be influenced by similar genes and physiological mechanisms (Amdam, Csondes, Fondrk, & Page, 2006; Amdam, Norberg, Fondrk, & Page, 2004; Evans & West‐Eberhard, 1970; Hunt et al., 2007; Linksvayer & Wade, 2005; Page & Amdam, 2007; West‐Eberhard, 1978, 1987).

The maternal heterochrony hypothesis proposes that the evolutionary origin of sibling care results from a condition‐dependent shift in the timing of expression of maternal care genes (Linksvayer & Wade, 2005). Lower quality, less fertile females express care behaviors toward siblings before, or instead of, reproducing, while higher quality, fully fertile females express care postreproductively toward offspring. At the evolutionary origin of sibling care, before the evolution of discrete queen and worker castes, sibling care behavior is hypothesized to be phenotypically identical to maternal care behavior, and to share all underlying molecular genetic and physiological mechanisms (Linksvayer & Wade, 2005). After the evolution of queen–worker dimorphism, maternal care behavior expressed by queens and sibling care behavior expressed by workers are expected to diverge, depending on natural history and the associated specific patterns of selection shaping queen and worker traits. However, even in lineages with strong queen–worker dimorphism, brood care behaviors expressed by queens (assuming queens still express maternal care at all) and workers are still expected to share some mechanistic underpinnings due to pleiotropy. Therefore, queen‐ and worker‐expressed care behaviors are predicted to be genetically and phenotypically correlated to some degree (Linksvayer & Wade, 2005). Such intercaste genetic correlations are expected to broadly affect the evolution of queen and worker traits, and may constrain the independent optimization of queen and worker traits (Holman, 2014; Holman, Linksvayer, & d'Ettorre, 2013; Pennell, Holman, Morrow, & Field, 2018).

The maternal heterochrony hypothesis makes two major types of predictions focused on the molecular genetic basis of trait expression and the quantitative genetic basis of trait variation, which can be tested with functional genetic (e.g., transcriptomic) and quantitative genetic empirical data, respectively (Linksvayer & Wade, 2005). First, the maternal heterochrony hypothesis predicts that maternal and sibling care have shared molecular and physiological mechanisms. The degree of overlap is expected to be highest in facultatively and primitively eusocial species, where there is little to no queen–worker dimorphism and maternal and sibling care behaviors are phenotypically identical. The overlap is predicted to be lowest in obligately eusocial species with strong queen–worker dimorphism (although caste antagonism, antagonistic selection between castes, likely prevents either caste from expressing its optimal caste‐specific phenotype; Linksvayer & Wade, 2005; Pennell et al., 2018). Transcriptomic studies in primitively eusocial vespid wasps, bumble bees, and facultatively eusocial carpenter bees that explored the overlap of transcriptome‐wide gene expression profiles between workers and queens engaged in maternal care provide preliminary support for these predictions (Rehan, Berens, & Toth, 2014; Toth et al., 2007, 2010; Woodard, Bloch, Band, & Robinson, 2014). To date, this prediction has not been tested in ants, which are characterized by obligate eusociality.

Second, the maternal heterochrony hypothesis predicts that there will be phenotypic and genetic correlations between maternal and sibling care (Linksvayer & Wade, 2005). The magnitude and sign of the genetic correlation are expected to depend on the degree of queen–worker dimorphism (Table 1). When there is little or no queen–worker dimorphism (e.g., in facultatively and primitively eusocial species), maternal and sibling care behaviors are more or less phenotypically identical and are expected to show a strong, positive genetic correlation (i.e., *r* ~ +1.0). In contrast, in obligately eusocial species with strong queen–worker dimorphism, selection acting to simultaneously optimize both queen and worker traits is predicted to cause a negative genetic correlation between queen and worker traits, as a result of trade‐offs caused by intercaste antagonistic pleiotropy (Linksvayer & Wade, 2005; Pennell et al., 2018). That is, alleles that positively affect both queen and worker performance are expected to fix, alleles that negatively affect both queen and worker performance are expected to be lost, and only alleles that have opposing effects on queen and worker performance are expected to remain segregating and contributing to observed genetic correlations (Linksvayer & Wade, 2005). As far as we know, these predictions have not been empirically tested in any species, even though they are also key to hypotheses regarding the evolution of intercaste correlations and caste antagonism (Holman, 2014; Pennell et al., 2018), but see (Holman et al., 2013).

**Table 1:** Predicted directions of genetic and environmental correlations between maternal and sibling care when maternal and sibling care have high versus low similarity, corresponding to a relatively low versus high degree of queen–worker dimorphism

	Maternal and sibling care similarity	
	High	Low
Genetic	+	−
Environmental	+	+

Here, we estimated the phenotypic correlation between queen and worker care behaviors in the black garden ant *Lasius niger*, as a first exploration of the quantitative genetic predictions of the maternal heterochrony hypothesis and also to test whether we find evidence for intercaste correlations for brood care, as predicted by the intralocus caste antagonism framework (Pennell et al., 2018). In many ant species including *L*. *niger*, after mating, a single queen excavates a chamber, seals herself in, lays a first clutch of eggs, and independently cares for this first batch of larvae using fat reserves and wing musculature (Keller & Passera, 1989; Sommer & Holldobler, 1995). This first brood develops as workers, often called “nanitic” workers because of their small size, that exit the nest chamber to collect food and care for the second brood. Subsequently, the workforce and brood number rapidly grow from a few to hundreds, and care behavior is expected to transition from individuals (i.e., the queen or nanitic workers) providing all necessary care serially to a small number of larvae, to many workers providing care in parallel to many larvae, with the potential for worker specialization on certain aspects of brood care (e.g., caring for a certain larval stage; Walsh, Warner, Kase, Cushing, & Linksvayer, 2018), as well as assembly‐line dynamics (Anderson & Ratnieks, 1999; Hart, Anderson, & Ratnieks, 2002; Jeanne, 1986, 1999; Johnson & Linksvayer, 2010; Seeley, 1995).

Based on the maternal heterochrony hypothesis and hypotheses regarding the evolution of intercaste correlations (Holman, 2014; Holman et al., 2013; Linksvayer & Wade, 2005; Pennell et al., 2018), we make the following predictions: (a) If queen and worker nursing behaviors are genetically correlated due to pleiotropy, queen and worker nursing behaviors should also be phenotypically correlated; (b) If our measure of brood care is tied to colony fitness, we expect to find a positive relationship between brood care and colony productivity. We tested these predictions by measuring colony productivity and the nursing behavior of *L*. *niger* queens and first and later generation workers.

## 2 MATERIALS AND METHODS

### 2.1 Colony collection and setup

We collected recently mated *L. niger* queens during their nuptial flight in Paris, France in August 2012. We placed individual queens in glass tubes (length 15 cm, diameter 1.5 cm) plugged with a cotton plug. The bottom half of the tube was filled with water and plugged with cotton to provide the queen with water. We did not give the queens access to food because in nature they survive off their internal reserves until their first workers begin to forage. We maintained queens in the dark and at room temperature (ranging between 17 and 24°C).

We monitored each queen for egg‐laying and excluded queens that did not lay eggs by September 15th. After the first worker eclosed, we opened the tubes by removing the cotton plug and placed the tubes in individual plastic boxes (10 × 15 × 4 cm). We fed the colonies two times per week with honey and freshly frozen adult fruit flies (*Drosophila melanogaster*) and maintained them under an inverted photoperiod. We conducted all experiments during the day (which was night time for the ants) under red light to minimize disturbance.

### 2.2 Brood retrieving tests

#### 2.2.1 Queens

**Figure 1:**
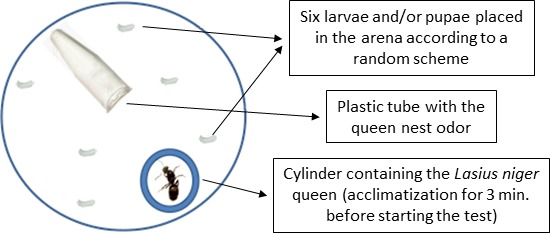
Schematic showing the experimental setup. We inserted in the Petri dish the queen or three workers separated by a plastic cylinder, six brood items, and the small plastic tube the ants used as a nest

Before starting the behavioral assays, we monitored the incipient colonies for the presence of at least six brood items (medium or large larvae and/or pupae). We inserted an Eppendorf tube (1.5 ml, without the cap and containing a small piece of humid cotton at the bottom) into the queen nest (the glass tube) and left it there for 3 days. In this way, the tube would get the nest odor and the queen could familiarize with it. On the fourth day, we placed the queen, six brood items (larvae and/or pupae), and the Eppendorf tube bearing the nest odor into a neutral circular arena (plastic Petri dishes, 50 mm diameter; Figure 1). We used a small paintbrush to manipulate brood items. To allow the queen to acclimatize to the arena, we placed the queen in a plastic cylinder in the arena for three minutes before beginning the assay. The arena was likely perceived by the queen as a novel environment and the Eppendorf tube as her nest. Therefore, the queen was expected to retrieve the brood items and bring them back to the tube.

We used Etholog 2.25 (Ottoni, 2000) to record the first contact with brood, the number of brood items retrieved within 10 min, and the duration of the total retrieving task. Differences between individuals in brood retrieval could be due to differences in nest mate recognition ability if individuals are more likely to retrieve brood from their own nest. Therefore, each individual was tested two times: once with brood from her own colony and once with brood from a conspecific colony. We conducted the two trials 20 min apart and randomized the order. During the 20‐min break, we placed the queens back in their own nest tube. We tested a total of 79 queens between October and November 2012.

#### 2.2.2 Workers

We monitored the colonies daily for the eclosion of the first generation of workers and marked each individual with a dot of paint on the thorax shortly after eclosion. We conducted the brood retrieval assay on workers when they were about 30 days old as prior to 30 days workers usually are not motivated to explore an unfamiliar arena. We tested worker brood retrieval identically to queen brood retrieval, although we tested three workers together. We used three workers (a) to imitate realistic social conditions and (b) to increase the likelihood of brood retrieval by workers, which may vary in their readiness to perform the task.

As for queens, we tested each group of workers two times, once with their own brood items and once with conspecific brood. We tested 67 groups of three‐first generation workers between November 2012 and February 2013. After the assays, we placed the tested workers back into their own nests.

Starting August 2013, we monitored the colonies daily for the eclosion of later generation workers. We marked the workers after eclosion and tested them after 30 days following the same procedure as the first generation workers. We tested 50 groups of three later generation workers between September and October 2013.

#### 2.2.3 Colony productivity

To test whether the brood retrieval of queens or workers was correlated with colony fitness, we measured the productivity of each colony by counting the number of workers and brood items present in the nest. We quantified productivity twice, once after each round of worker brood retrieval assays was completed.

### 2.3 Statistical analysis

We performed all statistical analyses in R version 3.4.1 (R Core Team, 2014). Retrieval rates (number of brood items retrieved divided by the duration of the retrieving task) were not normally distributed (Shapiro–Wilk test; *W *=* *0.964, *p *<* *0.001). Therefore, we used nonparametric Wilcoxon signed‐rank tests to examine whether retrieval rates were different when individuals retrieved their own brood versus brood from another colony. We used a nonparametric Kruskal–Wallis test when comparing the average retrieval rates of queens and workers. We used Spearman rank correlations when testing for correlations between retrieval rates of queens and workers. We confirmed the results of the Spearman rank correlations with generalized linear mixed models (GLMMs) for queen or worker retrieval rate and included brood identity (same colony of a different colony) as a fixed effect and colony identity as a random effect (see Supporting information Data S1, S2 and S3 for details). Finally, we fit GLMMs for colony productivity using the package MASS (Venables & Ripley, 2002). We included retrieval rates as a fixed effect and colony identity as a random effect. Because the productivity data were overdispersed (test for overdispersion: *W *=* *8.758, *p *<* *0.001), we used quasipoisson distributions in the GLMMs. We generated all plots using the package ggplot2 (Wickham, 2009).

## 3 RESULTS

Queens and workers showed no difference in their retrieval rates of same colony versus different colony brood (Wilcoxon signed‐rank test; queens: *W *=* *1,991, *p *=* *0.5027; first generation of workers: *W *=* *1,733, *p *=* *0.5152; later generations of workers: *W *=* *1,284, *p *=* *0.8146), suggesting nestmate recognition did not play a role in the retrieval assays. Therefore, we did not distinguish between brood types for the subsequent analyses.

**Figure 2:**
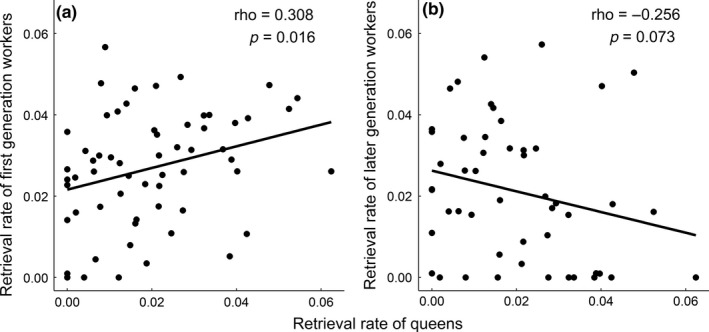
Scatterplot showing the correlation between retrieval rates (number of retrievals per second) of queens and first generation workers

First generation workers retrieved brood at a faster rate than queens but not later generation workers (Kruskal–Wallis; *χ* ^2^ = 8.963, *p *=* *0.0113; post hoc: queens and first generation workers: *p *=* *0.013; queens and later generation workers: *p *=* *0.870; first and later generation workers: *p *=* *0.073). Furthermore, the retrieval rates of queens and first generation workers were positively correlated (Spearman rank; *ρ* = 0.3080, *p *=* *0.0157; Figure 2), while the retrieval rates of queens and later generation workers and first and later generation workers were not correlated (queens and later generation workers: *ρ* = −0.2556, *p *=* *0.0733; first and later generation workers: *ρ* = −0.0577, *p *=* *0.6906).

**Figure 3:**
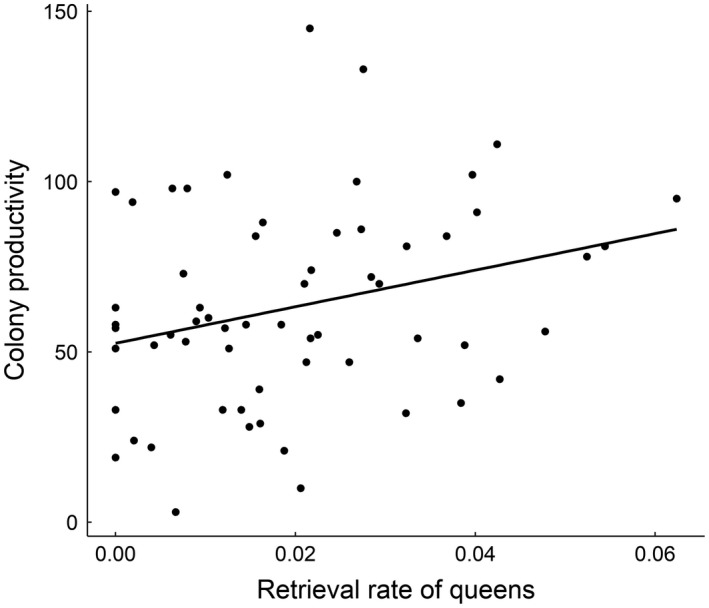
A scatterplot showing the colony productivity (total number of workers, larvae, and pupae in the colony) at the second census and the retrieval rate of queens (number of retrievals per second). The best fit line illustrates the relationship

At the first census, the retrieval rates of neither queens nor first generation workers had a significant effect on total colony productivity (GLMM: queens: *χ* ^2^ = 1.8289, *df* = 1, *p *=* *0.3212; workers: *χ* ^2^ = 1.0878, *df* = 1, *p *=* *0.4443), defined as the number of workers, larvae, and pupae in the colony. At the second census, the retrieval rate of queens was significantly positively associated with colony productivity (*χ* ^2^ = 62.812, *df* = 1, *p *=* *0.0215; Figure 3). The retrieval rate of workers was not associated with colony productivity (first generation workers: *χ* ^2^ = 24.803, *df* = 1, *p *=* *0.1581; later generation workers: *χ* ^2^ = 34.703, *df* = 1, *p *=* *0.0553). Colony productivity at the first census was significantly positively associated with colony productivity at the second census (*χ* ^2 ^= 73.024, *df* = 1, *p *=* *0.0132).

## 4 DISCUSSION

The evolution of eusociality is considered a major transition in evolution (Maynard Smith & Szathmáry, 1995). Sibling care, one of the defining features of eusociality, is hypothesized to have evolved via heterochronic changes in the expression of genes involved in maternal care. Here, we provide the first exploration in any species of the predictions made by the maternal heterochrony hypothesis regarding the quantitative genetic basis of trait variation. Specifically, the maternal heterochrony hypothesis predicts that maternal and sibling care will be phenotypically correlated due to shared genetic mechanisms underlying the two traits. In accordance with this prediction, we found that the brood retrieval rate, a component and proxy of care behavior, of *L*. *niger* queens was correlated with the retrieval rate of workers. Furthermore, we found that the retrieval rate of queens was positively correlated with colony productivity at the second census, suggesting natural selection is acting on maternal care. Although the retrieval rate of workers was not correlated with colony productivity, it is possible that worker retrieval rate is linked to colony productivity at larger colony sizes. While we tested the predictions of the maternal heterochrony hypothesis in only one species, these results support the idea of a shared genetic basis between maternal and sibling care and queen and worker traits more generally, which has implications for the hypothesized link between maternal care and the evolution of eusociality.

Interestingly, we found that the retrieval rate of queens was correlated with the retrieval rate of first generation but not later generation workers and that this correlation was positive. According to the maternal heterochrony hypothesis, the correlation between queen and worker traits is expected to become increasingly negative as queen–worker dimorphism increases because queen and worker traits cannot be both simultaneously optimized (Linksvayer & Wade, 2005) (Table 1). Therefore, assuming that maternal and sibling care behaviors are distinct to some degree, we would expect maternal and sibling care to be negatively correlated across all generations of workers in *L*. *niger*. However, we found a positive correlation between the brood care of queens and first generation workers (*ρ* = 0.3080, *p *=* *0.0157), and no significant correlation between the brood care of queens and later generation workers.

The discrepancy between the observed phenotypic correlation between queen and first generation versus later generation workers could be the result of maternal care being more similar to the care provided by first generation workers than the care provided by later generation workers. That is, if these two behaviors are very similar (as in the situation where there is little to no queen–worker dimorphism), we expect this correlation to be positive. The social environment experienced by the queen and the first generation workers is very different than the environment experienced by subsequent generations of workers, and indeed, the types of care behaviors performed may differ. Queens and first generation workers care for, and have to respond to, only a small number of developing larvae while subsequent generations of workers care for many more larvae. When colony size is small, colonies are not “fully functional” as the per capita work is expected to be linear in small colonies but nonlinear in large colonies (Jeanne, 1986, 1999; Johnson & Linksvayer, 2010). The transition from linear to nonlinear output is driven by the benefits of worker specialization and complex organization. For example, Jeanne (1999) found that *Polybia occidentalis* workers exhibit “assembly line dynamics” (Johnson & Linksvayer, 2010) wherein workers specialize on one of many different tasks and each worker requires the help of workers specialized on the other tasks. Colonies suffer a time delay if one of the tasks is performed more slowly than the others. Jeanne found that it requires at least 50 workers for the benefit of worker specialization to outweigh the time delay costs. Therefore, it is unlikely that workers in small *L*.* niger* colonies specialize on specific tasks and, similar to queens, must perform all necessary brood care behaviors (i.e., nursing, grooming, and carrying) on larvae of all developmental stages. In larger colonies, it is possible that *L*. *niger* workers specialize on caring for a subset of larval developmental stages or on performing a subset of brood care behaviors (Walsh et al., 2018).

Indeed, we know that first generation workers are not only morphologically distinct from postnanitic workers (first generation, nanitic workers can be as small as half the size of normal workers; Porter & Tschinkel, 1986), but are behaviorally distinct as well. For example, fire ant (*Solenopsis invicta*) nanitic workers are more efficient at rearing brood than larger sized workers (Porter & Tschinkel, 1986) while *Ectatomma tuberculatum* nanitic workers are less efficient at capturing prey than regular sized workers (Dejean & Lachaud, 1992). Overall, it is likely that the care provided by first generation workers is very different than the care provided by subsequent generations of workers and could therefore be considered as two different traits. This could explain the observed difference in the correlation with queen behavior.

We also cannot rule out the possibility that the observed phenotypic correlation between the retrieval rates of queens and first generation workers was due to shared environmental and not necessarily genetic factors. For example, it is possible that queens in good condition (e.g., with high fat reserves) are better able to care for the developing first generation workers. *L*. *niger* queens feed the developing nanitic workers using their own fat reserves and flight musculature (Keller & Passera, 1989; Sommer & Holldobler, 1995). Therefore, larger, better‐fed queens can likely produce higher quality nanitic workers, which in turn might have higher retrieval ability, resulting in a positive phenotypic correlation between queen and worker retrieval rates. However, we would expect high‐quality queens to also have high‐quality later generation workers due to the quality of care provided by first generation workers, but we observed this positive correlation in only the first generation of workers. Additional experiments are necessary to completely rule out the role of shared environmental factors.

Overall, our study provides the first test of the predictions of the maternal heterochrony hypothesis regarding the quantitative genetic basis of trait variation and provides support for the role of intercaste correlations and caste antagonism in the evolution of queen and worker traits in eusocial insects. Future experiments should test for phenotypic and genetic correlations between maternal and sibling care across independent origins of eusociality.

## AUTHORS’ CONTRIBUTIONS

TAL and PdE conceived the study; PdE and TAL designed the experiments; LS and PdE performed the methodology; LS performed the experiments; JTW analyzed the data; JTW wrote the original draft; JTW, TAL, and PdE reviewed and edited the draft; PdE and TAL supervised the project; and PdE and TAL acquired funding for the project.

## DATA ACCESSIBILITY

The data supporting this study and all analyses are available on Dryad: https://doi.org/10.5061/dryad.qm114cj
